# Temperature-Responsive Hybrid Composite with Zero Temperature Coefficient of Resistance for Wearable Thermotherapy Pads

**DOI:** 10.3390/mi16010108

**Published:** 2025-01-19

**Authors:** Ji-Yoon Ahn, Dong-Kwan Lee, Min-Gi Kim, Won-Jin Kim, Sung-Hoon Park

**Affiliations:** Department of Mechanical Engineering, Soongsil University, 369 Sangdo-ro, Dongjak-Gu, Seoul 06978, Republic of Korea; a024679@naver.com (J.-Y.A.); kd2890@naver.com (D.-K.L.); kmg4694@naver.com (M.-G.K.); dnjswls0214@gmail.com (W.-J.K.)

**Keywords:** carbon-based polymer composite, Joule heating, zero TCR, thermotherapy pad

## Abstract

Carbon-based polymer composites are widely used in wearable devices due to their exceptional electrical conductivity and flexibility. However, their temperature-dependent resistance variations pose significant challenges to device safety and performance. A negative temperature coefficient (NTC) can lead to overcurrent risks, while a positive temperature coefficient (PTC) compromises accuracy. In this study, we present a novel hybrid composite combining carbon nanotubes (CNTs) with NTC properties and carbon black (CB) with PTC properties to achieve a near-zero temperature coefficient of resistance (TCR) at an optimal ratio. This innovation enhances the safety and reliability of carbon-based polymer composites for wearable heating applications. Furthermore, a thermochromic pigment layer is integrated into the hybrid composite, enabling visual temperature indication across three distinct zones. This bilayer structure not only addresses the TCR challenge but also provides real-time, user-friendly temperature monitoring. The resulting composite demonstrates consistent performance and high precision under diverse heating conditions, making it ideal for wearable thermotherapy pads. This study highlights a significant advancement in developing multifunctional, temperature-responsive materials, offering a promising solution for safer and more controllable wearable devices.

## 1. Introduction

Wearable devices have gained significant attention for their ability to monitor user conditions in real-time while ensuring portability [1,2]. Flexible and wearable electronic devices, attachable to clothing or directly to the human body, support various activities such as personal health monitoring, human–machine interactions, and motion tracking [3,4,5,6,7]. Among these, flexible heaters utilizing Joule heating are integral to heated garments [8,9,10] and wearable thermotherapy systems [10,11,12]. Thermotherapy is a treatment that applies heat to specific body parts to alleviate injuries or symptoms and often involves the use of heating pads. The effectiveness of heat-based therapies has been widely reported in various domains [13,14,15,16], including arthritis [17,18] and dermatological conditions [19,20]. However, existing heaters, which are primarily composed of rigid [18] and planar components, lack flexibility and scalability. Commercial thermal therapy pads often offer only basic on/off functionalities without temperature control. Therefore, flexible and controllable carbon-based polymer composites have been explored for application in thermal therapy pads [21,22,23,24].

Several types of carbon nanofillers with different characteristics are used in carbon-based composites [25,26,27,28]. Among them, carbon black (CB), which is characterized by its spherical morphology resembling grape clusters and aggregation sizes ranging from tens to hundreds of nanometers, is widely employed to modify the mechanical and electrical properties of composites [29,30]. Carbon nanotubes (CNTs), on the other hand, exhibit exceptional mechanical and electrical properties due to their cylindrical molecular structure with high aspect ratios. CNTs offer superior thermal conductivity (6600 W m⁻¹K⁻¹), electrical conductivity (10⁶ S m⁻¹), and low mass density, enabling high conductivity in polymer matrices even at minimal filler fractions [30,31,32].

**Figure 1:**
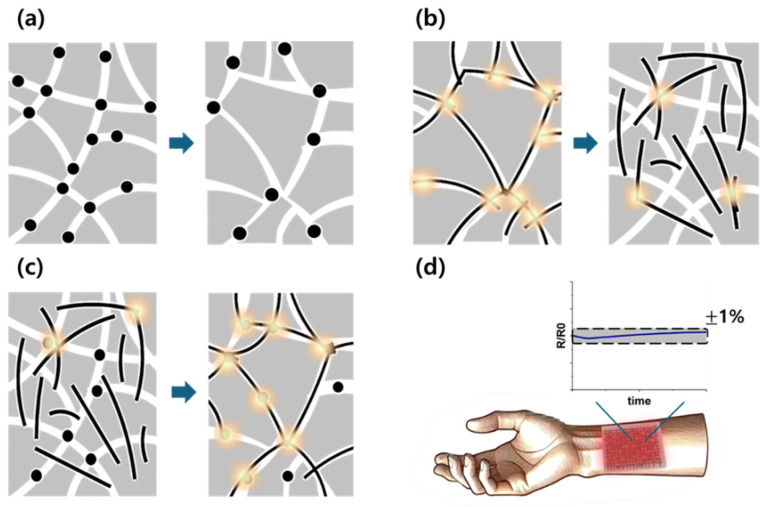
Schematic illustrations depicting the mechanism of resistance change during Joule heating due to thermal expansion: (**a**) PDMS-CB composite, (**b**) PDMS-CNT composite, and (**c**) PDMS-CNT, CB composite. (**d**) Temperature-detectable Z-TCR composite used for thermal therapy.

Carbon-based polymer composites exhibit exceptional properties that make them highly suitable for applications in wearable devices. However, to ensure their safe use as heating elements, addressing the issue of their temperature coefficient of resistance (TCR) is essential. The TCR results from changes in the number of conductive pathways within the polymer matrix driven by thermal expansion during Joule heating or exposure to external temperature variations [33,34,35], as shown in Figure 1a,b. These changes can result in various challenges that significantly affect the performance and safety of materials. When the resistance increases with increasing temperature, also known as the positive temperature coefficient (PTC), irregular structural changes during the heating and cooling cycles lead to poor reproducibility and a substantial increase in electrical resistance, making it difficult to achieve specific target temperatures [36,37]. Conversely, when the resistance decreases with increasing temperature, a behavior referred to as a negative temperature coefficient (NTC) and excessive current flow may occur, causing overheating or damage to the surrounding circuits. In cases where the material is in direct contact with the body, it can pose a serious risk of burns [37,38,39]. To ensure the safe and reliable application of carbon-based polymer composites in wearable heating devices, achieving zero-TCR (Z-TCR) is critical.

In addition to addressing the TCR issue, carbon-based polymer composites must incorporate a user-friendly system for temperature monitoring to ensure their safe use in wearable heaters. As wearable heating devices are often in direct contact with the body, preventing burns during use is of critical importance [40,41,42]. A system capable of detecting temperature changes is essential for mitigating the risk of burns, particularly low-temperature burns, which can occur without the user’s awareness [43,44]. Therefore, for the safe operation of heating pads, integrating a reliable temperature-indicating function is indispensable.

Extensive studies have been conducted to address these challenges. Zhu et al. developed fiber-shaped strain sensors with a near-zero TCR by incorporating GNP/CNT hybrid fillers into silicone elastomers [45]. Kim et al. fabricated flexible composites with micropatterned thermochromic displays to provide a visual temperature alert [21]. Laukkanen et al. introduced a sustainable cross-linked poly cyclohexyl urethane (PCVU) substrate with silver nanowires for flexible, stretchable transparent conducting electrode (TCE)-based strain sensors and heaters [46], and Reddy et al. developed a transparent conductive electrode (TCE) with an electro-spun polyvinyl alcohol (PVA) nanofiber mat sprayed with silver nanowires, which was applied to a wearable heating sensor [47]. However, to date, no study has simultaneously addressed the TCR issue in carbon-based polymer composites while enabling users to visually monitor temperature changes during thermal therapy.

This study aims to develop a bilayer PDMS-CNT and CB hybrid composite with a near-zero TCR, enabling visual temperature monitoring for wearable heating applications. The unique characteristics of carbon nanomaterials during Joule heating were analyzed to fabricate a hybrid composite capable of achieving a near-zero TCR. To ensure safe and precise applications in thermal therapy, a thermochromic pigment layer was integrated into the upper surface of the composite, as illustrated in Figure 1d. This layer provides a visual temperature indication by changing the color at specific temperature thresholds. The resulting carbon-based polymer bilayer composite offered reliable temperature control and enhanced safety, making it highly suitable for applications requiring both temperature monitoring and stability.

## 2. Experimental

### 2.1. Materials

CNTs, CB, a thermochromic pigment, and polydimethylsiloxane (PDMS) were used for the fabrication of temperature-detectable bilayer conductive composites. One-dimensional (1D) filler CNTs with an average diameter of 5 nm and length ranging from 50 μm to 150 μm were purchased from JEIO (Incheon, Gyeonggi-do, Republic of Korea). The zero-dimensional (0D) filler CB, with an average diameter of 34 nm and a true density of 1.7 g/m^3^, was purchased from Mitsubishi Chemical Corporation (Tokyo, Japan). The base polymer matrix of polydimethylsiloxane (PDMS; Sylgard 184) was purchased from Dow Corning (Midland, MI, USA). Thermochromic pigment particles (pink and green) with average diameters ranging from 1 to 5 µm were purchased from Nano I & C (Changwon-si, Republic of Korea).

### 2.2. Fabrication of Hybrid Bilayer Composite

First, the PDMS used to fabricate the bilayer composite was prepared by mixing the base and curing agent in a 10:1 ratio.

To fabricate the upper layer of the bilayer structure, two different colors of the thermochromic pigment were mixed with PDMS using a paste mixer (Daehwa, Seoul, Republic of Korea). The mixture was first processed at 500 rpm for 30 s and subsequently at 1500 rpm for 90 s. The resulting mixture was spread onto a metal mold with a thickness of 0.5 mm and pressed using a hot press (Qmesys Inc., Gyeonggi-do, Republic of Korea) at 35 °C and 15 MPa for 9 h to achieve a semi-cured state.

**Figure 2:**
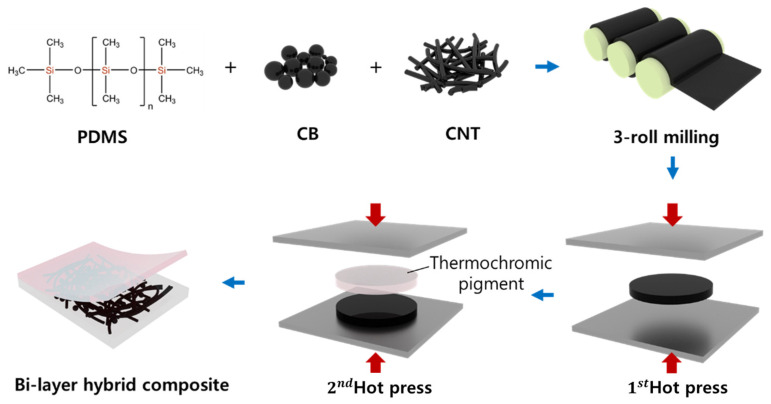
Fabrication of bilayer hybrid composites with carbon nanofillers and thermochromic pigments.

For the lower layer, PDMS was combined with the CNT and CB and mixed using a paste mixer at 500 rpm for 30 s, followed by 1500 rpm for 90 s. The mixture was processed using a three-roll milling machine (Intech, Gyeonggi-do, Republic of Korea) for 5 min to achieve uniform filler dispersion. The prepared composite was then spread onto a metal mold to a thickness of 1 mm. The semi-cured upper layer was subsequently attached to the top of the uncured lower layer, and the assembly was hot-pressed at 35 °C and 15 MPa for 48 h to complete the curing process. The fabrication process is illustrated in Figure 2.

### 2.3. Characterization and Test Conditions

The thickness and morphology of the fabricated bilayer structure were analyzed by observing cross-sectional fracture surfaces. The composite was frozen using liquid nitrogen, fractured, and examined using a Gemini SEM 300 (ZEISS Inc., Land Baden-Württemberg, Germany) to assess the dispersion of the CNT and CB.

The electrical conductivity was measured by forming electrodes on the fabricated samples. Specimens with dimensions of 50 × 5 mm were prepared, and their surfaces were etched for 5 min using a UV-ozone cleaner (JSE Co., Seoul, Republic of Korea) to maximize the exposure of CNTs and facilitate electrode formation. Silver paste (Protavic, Daejeon, Republic of Korea) was applied at four equally spaced points on the specimen and cured in an oven at 80 °C for 1 h. Electrical resistance was measured using the four-point probe method with a multimeter (DMM 7510, Keithley, Cleveland, OH, USA) to eliminate contact resistance.

Joule heating was induced by applying voltage to the specimen to measure the change in electrical resistance with temperature variation. Specimens with dimensions of 80 × 40 mm were prepared and etched for 5 min using a UV-ozone cleaner, and silver paste was applied at both ends. The paste was then cured in an oven at 80 °C for 1 h. The areas with silver paste were fixed to copper electrodes at constant pressure, and a DC power supply (ODA Technology, Incheon, Republic of Korea) was connected to a digital power analyzer (WT310E, Yokogawa Test & Measurement Corporation, Tokyo, Japan) to induce Joule heating and measure the resistance. The temperature change in the composite owing to Joule heating was monitored using an infrared camera (HIKMICRO M11W; Hangzhou Micro Image Software Co., Ltd., Hangzhou, China).

Additionally, to evaluate the application of the bilayer composite as a heating pad, a specimen (80 × 40 mm) with silver paste was attached to copper tape. Joule heating was induced using a DC power supply, and the resulting temperature distribution was confirmed using an infrared camera.

## 3. Results and Discussion

### 3.1. Percolation Threshold of PDMS-CNT and PDMS-CB

For Joule heating to occur, the material must possess sufficient electrical conductivity to facilitate conductive pathways for current flow. Highly conductive fillers, when uniformly dispersed within polymeric materials, such as PDMS, which inherently have very low electrical conductivity, can significantly enhance the electrical conductivity of the composite. The critical filler content at which a composite exhibits electrical conductivity is referred to as the percolation threshold.

**Figure 3:**
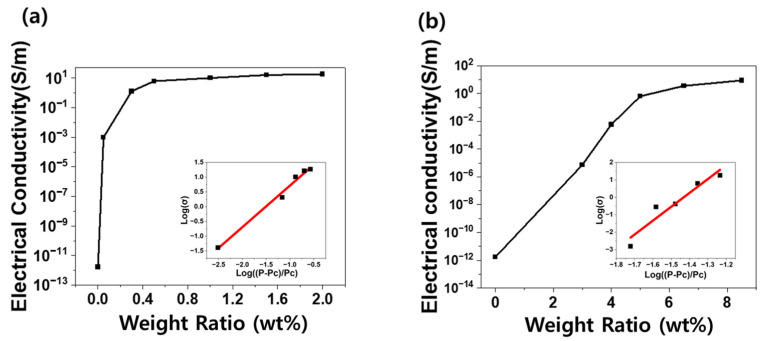
Percolation threshold graphs of (**a**) PDMS-CNT composites and (**b**) PDMS-CB composites.

As shown in Figure 3a, the percolation threshold for PDMS-CNT composites was observed to be 0.05 wt%, indicating the onset of electrical conductivity. Similarly, Figure 3b shows that the percolation threshold for the PDMS-CB composites was observed at 3 wt%. Theoretically, the electrical conductivity and percolation threshold can be calculated using the following equation [48]:

$$\sigma ={\sigma_{0}(\emptyset -\emptyset_{c})}^{t}$$

where σ is the electrical conductivity of the composite, $\sigma_{0}$ is a constant, ∅ is the filler content, $\emptyset_{c}$ is the percolation threshold, and t is the critical exponent. The critical exponent values for the PDMS-CNT and PDMS-CB composites were 1.40651 and 7.93971, respectively, which aligned with the trends observed in previous studies. The lower percolation threshold observed in the PDMS-CNT composites compared with that in the PDMS-CB composites was attributed to the morphological differences between the CNTs and CB. CNTs, as 1D materials, possess a high aspect ratio and a cylindrical structure that allow them to form conductive networks within the polymer matrix, even at relatively low filler contents. This unique structure enables the CNT to bridge larger gaps between particles within the polymer, resulting in a significantly lower percolation threshold. By contrast, CB is a 0D material with a spherical or quasispherodial shape. The formation of a conductive network in CB composites relies on the direct contact points between particles, requiring a closer spatial arrangement. Consequently, a higher filler content is required to achieve the percolation threshold in CB-based composites. These morphological differences underscore the importance of selecting an appropriate filler type and optimizing the dispersion of the fillers to meet the targeted performance requirements of the polymer nanocomposites. This optimization ensures enhanced electrical properties and facilitates the design of composites tailored for specific applications.

### 3.2. Analysis of Joule Heating and TCR Change

To analyze the temperature-dependent resistance changes in the CNT- and CB-based composites, a voltage was applied to induce Joule heating, and the resulting resistance changes over time were measured. As the temperature increased from room temperature to 120 °C, CNTs exhibited an NTC behavior, with resistance decreasing, while CBs showed a PTC behavior, with resistance increasing. These resistance changes in the flexible polymer nanocomposites were significantly influenced by the thermal expansion coefficient differences between the polymer and nanofillers. The thermal expansion coefficient of PDMS is approximately 3.2 × $10^{-4}K^{-1}$, whereas CNTs have a significantly lower coefficient of 1 × $10^{-6}K^{-1}$, and CBs exhibit a value of 9 × $10^{-6}K^{-1}$ [46,47]. This stark difference in the thermal expansion coefficients causes changes in the composite properties as the temperature increases, owing to Joule heating.

CNTs are cylindrical nanomaterials with a 1D honeycomb structure. As shown in Figure 1a, when a PDMS composite with dispersed CNTs undergoes thermal expansion, the center distance between the CNT nanoparticles increases. However, as the CNT strands are pushed around the expanding PDMS, they rearrange and align, increasing the number of contact points and conductive pathways [49]. This leads to a decrease in the resistance. In contrast, CB is a 0D nanomaterial that is isotropic when uniformly dispersed within PDMS, as shown in Figure 1b. When PDMS, in which CB is dispersed, undergoes thermal expansion, the distance between the dispersed CB nanoparticles increases, and the conductive pathway decreases, owing to the decrease in contact points between the nanofillers, resulting in an increase in resistance.

**Figure 4:**
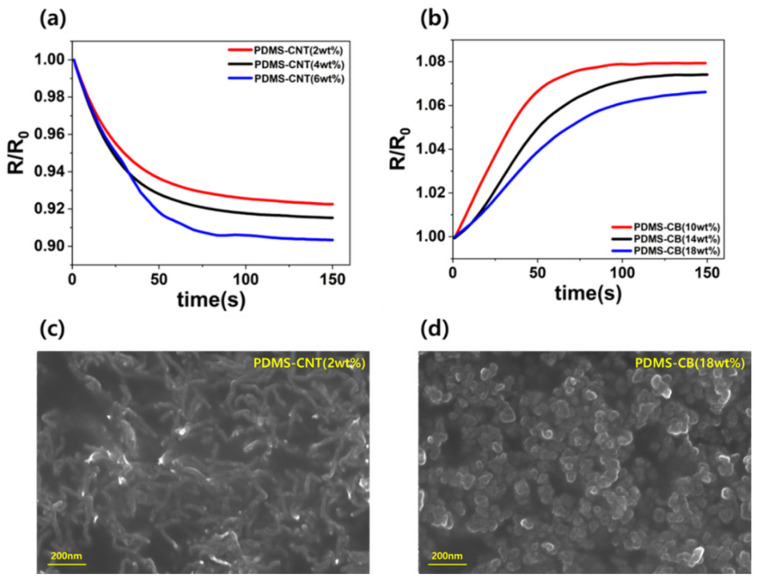
Normalized resistance graphs during Joule heating at 120 °C for 150 s: (**a**) PDMS-CNT composites and (**b**) PDMS-CB composites with varying filler content. The SEM images with 64k magnification of (**c**) PDMS-CNT-1.5 and (**d**) PDMS-CB-8.5.

Figure 4a,b show the resistance change rates of the CNT and CB composites measured over 150 s of Joule heating at different filler contents, showing opposite trends. CNT composites with 2 wt%, 4 wt%, and 6 wt% were compared. After 150 s, the resistance change rates are 7.7%, 8.5%, and 9.7%, respectively. A higher filler content resulted in higher resistance change rates owing to the increased formation of conductive pathways through thermal expansion. Although higher CNT content improves the electrical performance, it also increases the resistance variability under thermal conditions, as shown in Figure 4a. The CB composites at 14 wt%, 16 wt%, and 18 wt% were compared. After 150 s, the rates of resistance change were 7.9%, 7.4%, and 6.6%, respectively. A higher filler content decreased the resistance change rates owing to the reduced loss of conductive pathways during thermal expansion, as shown in Figure 4b. However, an excessive CB content may lead to increased brittleness, limiting its application as a flexible composite. These results underscore the need to balance the filler content for optimal performance while maintaining the flexibility and safety of the composite in thermal applications.

SEM analyses were performed on the PDMS-CNT (2 wt%) and PDMS-CB (18 wt%) composites. As shown in Figure 4c,d, the carbon nanofillers were uniformly dispersed within the polymer matrix to form a conductive path.

### 3.3. Analysis of CNT and CB Content of Z-TCR Hybrid Composite

Considering sufficient electrical conductivity and minimal resistance variation, the PDMS-CNT content in the hybrid composites was fixed at 2 wt%, whereas the CB content was adjusted to identify the composition that achieved a zero-temperature coefficient of resistance. As shown in Figure 4a,b, the CNTs exhibit NTC characteristics, whereas the CB demonstrate PTC behavior, showing opposing resistance trends. These contrasting properties of the two carbon nanomaterials were leveraged in experiments aimed at minimizing the TCR.

**Figure 5:**
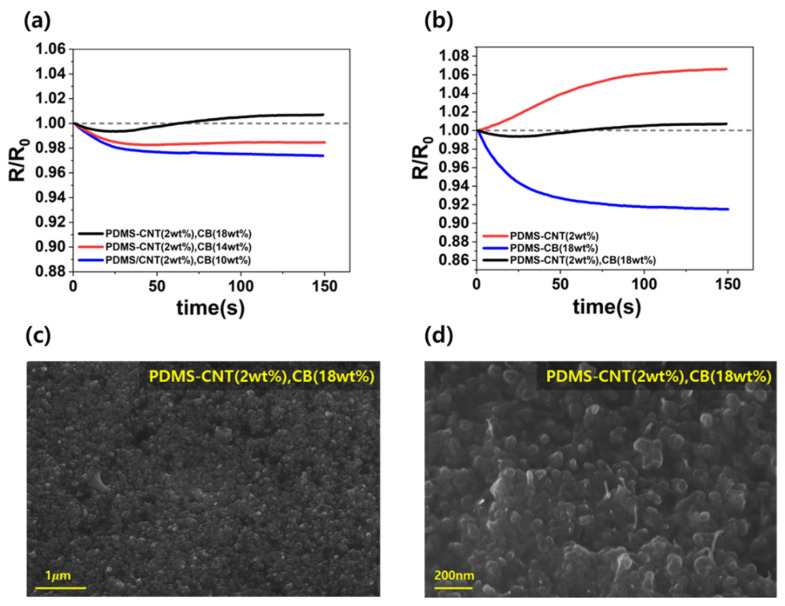
Normalized resistance graphs during Joule heating at 120 °C for 150 s: (**a**) graph showing the influence of CB as a PTC filler on PDMS-CNT composites, (**b**) PDMS-CNT(2 wt%), PDMS-CB(18 wt%), and PDMS-CNT(2 wt%), CB(18 wt%) composites. SEM images of PDMS-CNT(2 wt%), CB(18 wt%) composites with (**c**) 18.5k and (**d**) 64.6k magnification.

SEM analysis of the fabricated composite (PDMS-CNT-2 wt%, CB-18 wt%) confirm the uniform dispersion of carbon nanofillers throughout the polymer matrix, as shown in Figure 5c. At a higher magnification (Figure 5d), even relatively low CNT content is found to be uniformly distributed.

The resistance change rates of the hybrid composites were measured at varying CB contents (10 wt%, 14 wt%, and 18 wt%) while maintaining the CNT content at 2 wt%. For the composite with 10 wt% CB, the resistance change rate was observed to be −2.6%, demonstrating the retention of NTC behavior, a characteristic of CNTs. Increasing the CB content to 14 wt% resulted in a resistance change rate of −1.7%, still exhibiting NTC tendencies. However, at 18 wt% CB, the composite displayed a transition to PTC behavior after 62 s of heating, with resistance variation ranging between −0.7% and +0.6% during the heating process. By the end of the heating period, the rate of resistance change stabilized at +0.6%. This trend indicates that, as the CB content increases, the PTC behavior becomes more prominent, effectively reducing the overall resistance variability and achieving a near-zero TCR. For composites with CB content exceeding 18 wt%, the dominant PTC behavior would likely lead to increased resistance variability.

Figure 5b demonstrates the performance of the optimized hybrid composition (PDMS-CNT(2 wt%), CB(18 wt%), where resistance variation was maintained within a ±1% range. At 120 °C, the resistance change rate of this hybrid composite was 8.3% lower compared to PDMS-CNT(2 wt%) and 6% higher compared to PDMS-CB(18 wt%). As shown in Figure 1c, this improvement is attributed to the complementary effects of the NTC (CNT) and PTC (CB) properties. The interplay between the newly formed and diminished conductive pathways during thermal expansion ensures a balanced compensation. These findings highlight that a near-zero TCR can be achieved by appropriately mixing NTC and PTC carbon nanofillers at optimal ratios.

### 3.4. Bilayer Hybrid Composite Joule Heating Characteristics

**Figure 6:**
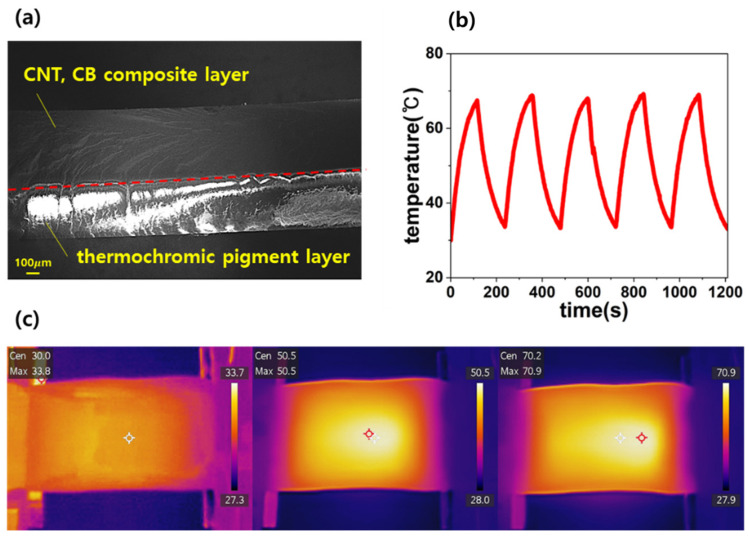
(**a**) SEM images at 43× magnification of PDMS-CNT(2 wt%), CB(18 wt%). (**b**) Repeated and rapid on–off thermal responses at an applied voltage of 8 V. (**c**) Infrared images taken during the repetition of the heating and cooling cycle.

To improve safety during heating, a PDMS-CNT (2 wt%) and CB (18 wt%) hybrid composite was integrated with a thermochromic pigment layer that visually signals temperature changes. The thermochromic pigments were a 1:1 mixture of two types: one that transitions to green at 40 °C and another that transitions to pink at 60 °C. Figure 6a shows an SEM image confirming the well-formed layered structure of the bilayer composite, with a distinct separation between the thermochromic pigment layer and the CNT-CB hybrid composite layer. The thermochromic pigment layer adheres securely to the carbon composite, ensuring that the heat generated by Joule heating in the composite layer is effectively transferred to the thermochromic layer.

To evaluate the response characteristics under repeated voltage application, a cyclic test was performed by alternating between the turn-on (8 V) and turn-off (0 V) states, each lasting for 120 s, resulting in a total cycle duration of 240 s. The test was repeated for five cycles. During the turn-on phase, the composite reached a peak temperature of 70.2 °C, while the minimum temperature during the turn-off phase was 30 °C. As shown in Figure 6b, the temperature profile remains consistent across all cycles, indicating that the heating and cooling behaviors of the composite are stable under repeated voltage application. This consistency demonstrates that the composite maintains a reliable heat output, making it suitable for wearable heating applications where stability is critical.

Figure 6c presents infrared (IR) images captured during the cyclic testing at three distinct temperatures: the minimum temperature (30 °C), an intermediate heating temperature (50.5 °C), and the maximum temperature (70.2 °C). The uniform heat distribution observed in these images confirms the dispersion of the conductive fillers within the composite. Moreover, the integration of the thermochromic pigment layer did not affect the original Joule heating characteristics of the composite, thereby ensuring its compatibility and functionality. These findings indicate that the bilayer hybrid composite is highly stable and suitable for wearable heating applications, ensuring user safety and reliable temperature control during prolonged use.

### 3.5. Verification of Safety and Application in Thermotherapy

**Figure 7:**
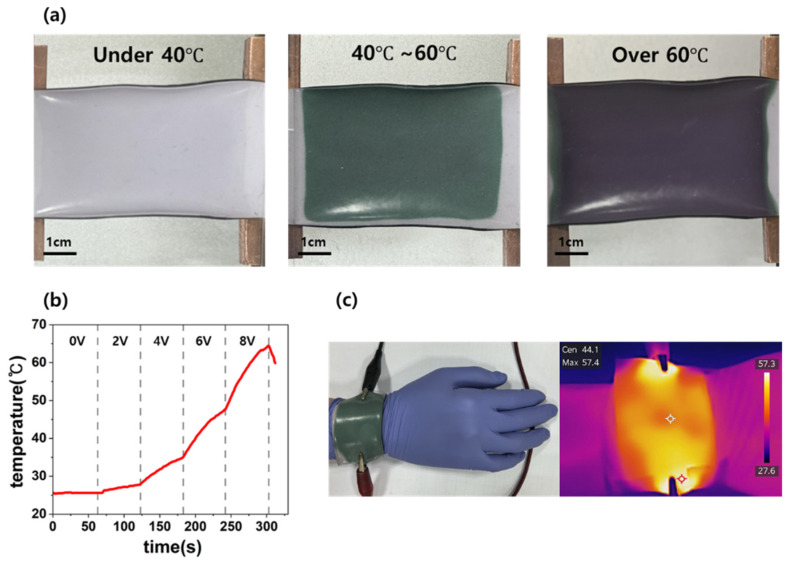
(**a**) Color changes in the bilayer composite across three temperature ranges based on the activation of thermochromic pigments. (**b**) Correlation between the applied voltage for Joule heating and the corresponding temperature increase. (**c**) Application of the hybrid bilayer composite for wrist thermotherapy.

The thermochromic pigment layer added to the PDMS-CNT (2 wt%) and CB (18 wt%) hybrid composites enhances the safety by visually indicating temperature changes. This layer operates by encapsulating a transparent liquid within microcapsules, which solidifies and displays color upon heating to specific thresholds. To ensure the reliable performance of the wearable heaters, thermochromic pigments capable of maintaining their properties after repeated heating and cooling cycles were selected. The pigments were designed to change color at specific temperatures: green at 40 °C and pink at 60 °C. Figure 7a illustrates the color transitions observed across three temperature ranges: below 40 °C, 40–60 °C, and above 60 °C. Below 40 °C, both pigments remained inactive, resulting in a light-pink shade close to the original base color. Between 40 °C and 60 °C, the green pigment was activated, turning the composite deep green. At temperatures exceeding 60 °C, both green and pink pigments were active, producing a purple shade.

The uniformity of color changes across the surface confirmed the consistency of the pigment dispersion, ensuring a reliable temperature indication. After heating, the composite reverted to its original light-pink shade upon cooling, demonstrating its reversibility and durability over repeated use. The heating performance of the bilayer hybrid composites was tested under applied voltages of 2 V, 4 V, 6 V, and 8 V. Voltages were incrementally applied in 2 V steps, with each voltage maintained for 60 s until reaching 8 V, during which the temperature changes in the composite were recorded. After 60 s of 2 V application, the composite reached 29.3 °C. At 4 V, the temperature rose to 38 °C, at 6 V to 51.8 °C, and at 8 V to 64.5 °C. As shown in Figure 7b, the temperature increases linearly with the voltage, allowing the determination of the voltage required to achieve a specific target temperature. Importantly, the activation of the thermochromic pigments during heating did not interfere with the Joule heating behavior of the composite, indicating the compatibility between the thermochromic layer and the carbon composite. Figure 7c illustrates the application of the hybrid composite as a temperature-sensing thermotherapy pad. Based on the heating data shown in Figure 7b, appropriate voltages were applied to achieve temperatures suitable for thermotherapy. Using an IR camera, uniform heat distribution and color changes were observed even when the composite was attached to a curved surface, such as a wrist. The thermochromic layer functioned reliably, enabling users to safely monitor temperature changes while maintaining the heating characteristics of the composite. These results highlight the potential of hybrid composites for thermotherapy applications, providing both safe and user-friendly temperature monitoring.

## 4. Conclusions

In this study, the temperature-dependent resistance characteristics of CNTs and CB were analyzed to develop hybrid composites with near-zero TCR. CNTs demonstrated NTC behavior, where the resistance decreased owing to realignment as the polymer matrix expanded during Joule heating. Conversely, CB exhibited a PTC, with the resistance increasing as the polymer matrix expansion reduced the number of contact points between the CB particles. By combining these opposing properties, complementary effects of CNTs and CBs were observed, resulting in reduced resistance variation. The optimal composition ratio for achieving the Z-TCR was determined. In addition, a thermochromic pigment layer that changed color across various temperature ranges was incorporated to create a bilayer composite. This layer enabled accurate temperature indication without compromising the functionality of the hybrid composite. The properties of the bilayer composite were maintained during repeated heating and cooling cycles, demonstrating reliable temperature control and stability.

By eliminating the resistance variation issues caused by Joule heating and integrating a temperature-indicating function, a safe and precise wearable thermotherapy pad was developed. This study provides a foundation for the application of carbon–polymer composites in wearable heaters across diverse fields where safety and precision are critical.

## Data Availability

The original contributions presented in this study are included in this article. Further inquiries can be directed to the corresponding author(s).
